# The impact of published guidance on trends in the pharmacological management of depression in children and adolescents- a whole population e-cohort data linkage study in Wales, UK

**DOI:** 10.1017/S0033291724002861

**Published:** 2024-12

**Authors:** A. Marchant, J. McGregor, M. Del Pozo Banos, K. Lloyd, D. Williams, A. Thapar, A. Watkins, A. John

**Affiliations:** 1 Swansea University Medical School, Swansea, UK; 2 Aneurin Bevan Health Board: NHS Wales Aneurin Bevan University Health Board, Gwent, UK; 3 Department - School of Medicine, Cardiff University, Cardiff, UK

**Keywords:** antidepressants, children, depression, policy, SSRIs, young people

## Abstract

**Background:**

This study evaluated the impact of 2015/2016 prescribing guidance on antidepressant prescribing choices in children.

**Methods:**

A retrospective e-cohort study of whole population routine electronic healthcare records was conducted. Poisson regression was undertaken to explore trends over time for depression, antidepressant prescribing, indications and secondary care contacts. Time trend analysis was conducted to assess the impact of guidance.

**Results:**

A total of 643 322 primary care patients in Wales UK, aged 6–17 years from 2010–2019 contributed 3 215 584 person-years of follow-up. Adjusted incidence of depression more than doubled (IRR for 2019 = 2.8 [2.5–3.2]) with similar trends seen for antidepressants. Fluoxetine was the most frequently prescribed first-line antidepressant. Citalopram comprised less than 5% of first prescriptions in younger children but 22.9% (95% CI 22.0–23.8; 95% CI 2533) in 16–17-year-olds. Approximately half of new antidepressant prescribing was associated with depression. Segmented regression analysis showed that prescriptions of ‘all’ antidepressants, Fluoxetine and Sertraline were increasing before the guidance. This upward trend flattened for both ‘all’ antidepressants and Fluoxetine and steepened for Sertraline. Citalopram prescribing was decreasing significantly pre guidance being issued with no significant change afterward.

**Conclusions:**

Targeted intervention is needed to address rising rates of depression in children. Practitioners are partially adhering to local and national guidance. The decision-making process behind prescribing choices is likely to be multi-factorial. Activities to support implementation of guidance should be adopted in relation to safety in prescribing of antidepressants in children including timely availability of talking therapies and specialist mental health services.

## Introduction

Rates of antidepressant prescribing for children and young people (CYP) have been rising in the UK since around 2005; with the most rapid increases in 15- to 17-year-old females (John et al., 2016a; Sarginson et al., 2017). Rates of probable mental disorders in children aged 7 to 16 years rose from 12% in 2017 to 17% in 2020 then stabilized in 2021 and 2022, in those aged 17 to 19 years they continued to rise from 10% In 2017 to 26% in 2022 (NHS Digital, 2022). Untreated, chronic depression in CYP may lead to both short and long-term adverse outcomes, such as poor social functioning, educational underachievement, substance use, self-harm, increased likelihood of co-morbidity, and being socio-economically disadvantaged in later-life (Kim-Cohen et al., 2003; Thapar, Collishaw, Pine, & Thapar, 2012; Thapar, Eyre, Patel, & Brent, 2022). Early detection, intervention, and management strategies, including psychological and pharmacological treatment options for depression in CYP are important to reduce long-term adverse outcomes.

Fluoxetine is the only licensed medication for CYP (ages 8+) for moderate to severe depression in the UK based on its efficacy and relative safety (Goodyer et al., 2007, 2008; Hetrick, Merry, McKenzie, Sindahl, & Proctor, 2007; March et al., 2004; Thapar et al., 2022). Evidence for Tricyclic antidepressants (TCAs) is less favorable (Hazell & Mirzaie, 2013). Treatment guidelines emphasize that antidepressants should only be prescribed by a specialist (NICE, 2019; Thapar et al., 2022). However, many primary care referrals to specialist Child and Adolescent Mental Health Services (CAMHS) in the UK are rejected and waiting times may result in ‘holding’ prescribing (Hinrichs, Owens, Dunn, & Goodyer, 2012; John et al., 2016a). Consequently, primary care may remain the most common source of care for CYP with mental health problems, independent of whether a GP feels an individual requires more specialist support.

Citalopram, an un-licensed medication for depression in this age group was the most prescribed antidepressant for CYP in Wales, UK (population 3 million) between 2003 and 2013 (John et al., 2016a, 2019). This ‘off-label’ prescribing of antidepressants is common across western countries and may reflect, in older adolescents, prescribing patterns in adults (Bachmann et al., 2016). Prescribing for indications outside of depression is also common with only around half of new antidepressant prescriptions in CYP associated with depression, with other indications including anxiety and pain (Grégoire & Finley, 2013; John et al., 2016a).

In response to safety and efficacy concerns and to improve the prescribing choices, local and national guidelines were published in 2015/16 in the UK (NICE, 2019; Welsh Government, 2015; WeMeRec, 2016). There are similar guidelines in many other countries. Most recommend a stepwise approach to treatment and to only use SSRIs for moderate-severe depression and to avoid using them alone as a treatment. A Welsh Health Circular (WHC; (Welsh Welsh Government, 2015)) was issued to GPs, specialist Child and Adolescent Mental Health Services (CAMHS) consultants, pediatricians and pharmacists in October 2015 by the Welsh Government in response to our previous work on prescribing for depression in Wales (John et al., 2016b). This circular emphasized Fluoxetine was the only effective, safe and licensed antidepressant to treat depression in CYP, whilst all other SSRIs (with particular emphasis on Citalopram) were unfavorable based on the balance of risks and benefits. Although it was recognized that specialists may sometimes decide to use these drugs in response to individual clinical need (Welsh Government, 2015). Further guidance was released in a bulletin issued in March 2016 by the Welsh Medicines Resource Centre (WeMeRec, 2016). This guidance advocated Fluoxetine, whilst highlighting that the risk-benefit ratio for all other SSRIs (including Citalopram), Venlafaxine and TCAs were unfavorable.

The aim of this study is to examine recent trends of depression and antidepressant prescribing over time including indications for use and whether prescriptions may have been initiated in secondary care. We evaluate whether relevant UK NICE, WHC and WeMeRec published guidelines have had any impact on prescribing choices.

## Methods

### Design

This is a retrospective e-cohort study.

### Data source

We linked data on an individual level via the Adolescent Mental Health Data Platform (ADP), an international data platform that supports mental health research in CYP. For our study, we used datasets from the SAIL Databank, a repository of routinely collected health and education datasets for the population of Wales (Ford et al., 2009; Lyons et al., 2009).

The following datasets were linked at patient level: Welsh Demographic Service (WDS); Welsh Index of Multiple Deprivation, containing deprivation score for all Lower Super Output Areas in Wales; General Practice Database (GPD), containing information for all GP interactions covering 79% of the Welsh population; Patient Episode Database for Wales (PEDW) containing data for all NHS Wales hospital admissions; Outpatients database (OPD) provides attendance information for all NHS Wales hospital outpatient appointments; Office of National Statistics (ONS) deaths register. Datasets are described in full at https://web.www.healthdatagateway.org/.

### Study population and setting

Individuals aged 6–17 years registered with SAIL supplying GPs for a minimum of 6 months from 1 January 2010–31 December 2019 were included in the study population.

Follow-up commenced at study onset, date of GP registration date plus 6 months to exclude the risk of retrospective recording or 6^th^ birthday whichever was the later. Data collection ended at age 18, date of leaving a SAIL supplying GP, date of death or study end, whichever was the earliest. Each individual could contribute more than one data period during the study as long as the above criteria were met.

### Measures

Demographic information (sex, age, and deprivation quintile) was collected at the onset of follow-up each year. Person time was calculated between the start and end dates for each year. Age was categorized into three groups 6–11 years, 12–14 years, and 15–17 years.

‘Depression was assessed using externally validated measures applied to GP data including both diagnosis and symptom codes as defined and validated previously (Cornish, John, Boyd, Tilling, & Macleod, 2016; John et al., 2016a). These code lists have been developed and validated using multiple possible algorithms and have been extensively utilized in previous research. For a full description see https://phenotypes.healthdatagateway.org/phenotypes/PH1114/version/2455/detail/'.

Incidence and prevalence of depression and antidepressant prescriptions were explored. Incidence was defined as no record in the previous 12 months. Prevalence was defined as any record of the above within a given year, independent of any previous events (John et al., 2016b). We explored ‘Treated’ events. This was defined as a diagnosis or symptom date with at least one antidepressant prescribing event within six months either side of the diagnosis or symptom event date.

We also explored antidepressant prescriptions in the population as a whole independent of diagnosis or indication. First ever incident prescriptions were divided by antidepressant type and proportions calculated to explore which antidepressants were being prescribed as first line antidepressant and which were most likely to be prescribed subsequently once another antidepressant has already been prescribed.

Antidepressants may be prescribed for a number of conditions other than depression. GP data does not explicitly list the indication for a prescription. We identified possible indications by temporal proximity within one year before and six months after annual incident antidepressant prescriptions. We firstly identified cases of depression, followed by anxiety and depression. For those without a record of depression/anxiety, we searched the GP records to identify the following probable indications: ‘pain’ (including neuropathic pain and headaches/migraine prophylaxis), enuresis and ‘other’, which included, attention deficit hyperactivity disorder (ADHD), conduct disorder, autism spectrum disorders (ASD), psychosis, eating disorders, sleep problems and irritable bowel syndrome. We further categorized ‘unknown’, where no apparent indication was established. No hierarchical system was applied meaning prescriptions could be linked to more than one category i.e. both ‘pain’ and ‘other’.

Prescribing data is only available in GPD but may have originated from secondary or tertiary care level settings and are simply prescribed by GPs. This could be following outpatient review or following hospital discharge note instructions. To gain an understanding of the source and origins of prescribing, we identified: Hospital admissions with a recording of an ICD10 code for a mental health diagnosis (F01-99); Outpatient appointments under a psychiatric or pediatric speciality; GP records with a read code for a mental health diagnosis/symptom. These were explored in the year preceding an incident prescription. We further categorized ‘primary care only’ and ‘unknown’ setting, for prescriptions with no record of a mental health event in any of the settings within the preceding 12 months.

### Statistical analysis

All statistical analyses were conducted using SPSS statistical software (version 20).

Poisson regression was undertaken to model the counts of the yearly rates for recorded depression and antidepressants, as a function of year, sex age group and deprivation. The significance of the variables in the Poisson regression modelling were assessed using Wald tests.

An interrupted time-series analysis was conducted to assess the significance based on quarterly data using segmented regression (Wagner, Soumerai, Zhang, & Ross-Degnan, 2002). Interruptions were set at time of publication of each of the three guidelines (NICE [March 2015], WHC [October 2015], WeMeRec [March 2016]). The analysis divides the time series into before and after the event of interest, and tested whether there was a significant step change or change in the slope in response to each intervention (Kendrick, Stuart, Newell, Geraghty, & Moore, 2015). We tested for autocorrelation in each model using the Durbin-Watson statistic (Durbin & Watson, 1971). No strong evidence for autocorrelation was found.

All confidence intervals (CIs) for proportions were estimated by the Wilson score method with continuity correction (Newcombe, 1998) and all CIs for rates were estimated using two-tailed mid-p exact CIs (assuming Poisson distribution) (Rothman & Boice, 1979).

## Results

### Study population

A total of 643 322 registered GP patients aged 6 to 17 years between 1 January 2010 and 31 December 2019 contributed 3 215 584 person years of follow-up data (online Supplementary Fig. S1). The mean follow-up time was 4.6 years.

### Depression

A total of 24 198 (3.8%) individuals had 26 282 new incident records of depression (Diagnoses: 7274 (1.1%) individuals, 7539 events; Symptoms: 19 657 (3.1%) individuals, 21 105 events). The proportion of depression incidences identified as ‘treated with an antidepressant’ were more than double for those with a diagnosis compared to those with depressive symptoms (Diagnoses: 52.7% [95% CI 51.2–54.3; *n* = 2955] Symptoms: 20.1% [95% CI 18.9–31.3; *n* = 4237).

#### Demographic variables

Females were more than twice as likely as males to have a record of depression. Incidence was highest in 15–17-year-olds and lowest in 6–11-year-olds. Incidence of depression increased with deprivation quintile (Table 1). Similar results were seen for prevalence (online Supplementary Table S1). Overall incidence and prevalence were higher for records of symptoms than diagnoses however demographic differences were comparable (online Supplementary Table S2).

**Table 1.** Incidence per 1000 PYAR (95% CI) and IRR^a^ (95% CI)^b^ of depression and antidepressant prescriptions in primary care

	Depression^c^		Antidepressant Prescriptions^d^	
	Incidence	IRR	Incidence	IRR
Year				
2010	4.6 (4.4–4.9)	Reference *p* < 0.001	4.6 (4.4–4.9)	Reference *p* < 0.001
2011	5.6 (5.3–5.8)	1.2 (1.0–1.4)	5.6 (5.3–5.8)	1.2 (1.0–1.3)
2012	6.1 (5.9–6.4)	1.3 (1.2–1.5)	6.1 (5.9–6.4)	1.2 (1.1–1.4)
2013	7.1 (6.8–7.4)	1.5 (1.3–1.7)	7.1 (6.8–7.4)	1.4 (1.3–1.6)
2014	7.4 (7.1–7.7)	1.6 (1.4–1.8)	7.4 (7.1–7.7)	1.5 (1.3–1.7)
2015	8.7 (8.4–9.1)	2.0 (1.7–2.2)	8.7 (8.4–9.1)	1.8 (1.6–2.0)
2016	8.4 (8.1–8.7)	1.9 (1.7–2.2)	8.4 (8.1–8.7)	1.6 (1.4–1.8)
2017	10.0 (9.6–10.3)	2.3 (2.1–2.6)	10.0 (9.6–10.3)	1.7 (1.6–1.9)
2018	11.4 (11.1–11.8)	2.7 (2.4–3.0)	11.4 (11.1–11.8)	1.9 (1.7–2.1)
2019	12.1 (11.7–12.5)	2.8 (2.5–3.2)	12.1 (11.7–12.5)	1.8 (1.6–2.0)
Sex				
Male	4.9 (4.8–5.0)	Reference *p* < 0.001	4.9 (4.8–5.0)	Reference *p* < 0.001
Female	11.7 (11.5–11.8)	2.4 (2.3–2.5)	11.7 (11.5–11.8)	2.5 (2.4–2.7)
Age group				
6–11 years	1.0 (0.9–1.0)	Reference *p* < 0.001	1.0 (0.9–1.0)	Reference *p* < 0.001
12–14 years	10.0 (9.8–10.2)	10.8 (9.7–12.1)	10.0 (9.8–10.2)	10.2 (8.5–12.2)
15–17 years	24.2 (23.9–24.6)	26.4 (23.6–29.6)	24.2 (23.9–24.6)	58.1 (48.8–69.3)
Deprivation				
Least deprived	6.9 (6.7–7.1)	Reference *p* < 0.001	6.9 (6.7–7.1)	Reference *p* < 0.001
2	7.3 (7.1–7.6)	1.1 (1.0–1.2)	7.3 (7.1–7.6)	1.0 (1.0–1.1)
3	7.9 (7.7–8.2)	1.2 (1.1–1.3)	7.9 (7.7–8.2)	1.1 (1.0–1.2)
4	8.5 (8.3–8.8)	1.3 (1.2–1.4)	8.5 (8.3–8.8)	1.2 (1.2–1.3)
Most deprived	9.7 (9.5–9.9)	1.5 (1.4–1.6)	9.7 (9.5–9.9)	1.4 (1.3–1.5)

a Adjusted for calendar year, age, sex and deprivation.

b Based on Wald test.

c Depression and symptoms combined.

d All antidepressant prescriptions with no breakdown or exclusion by antidepressant type.

#### Trends over time

**Figure 1.**
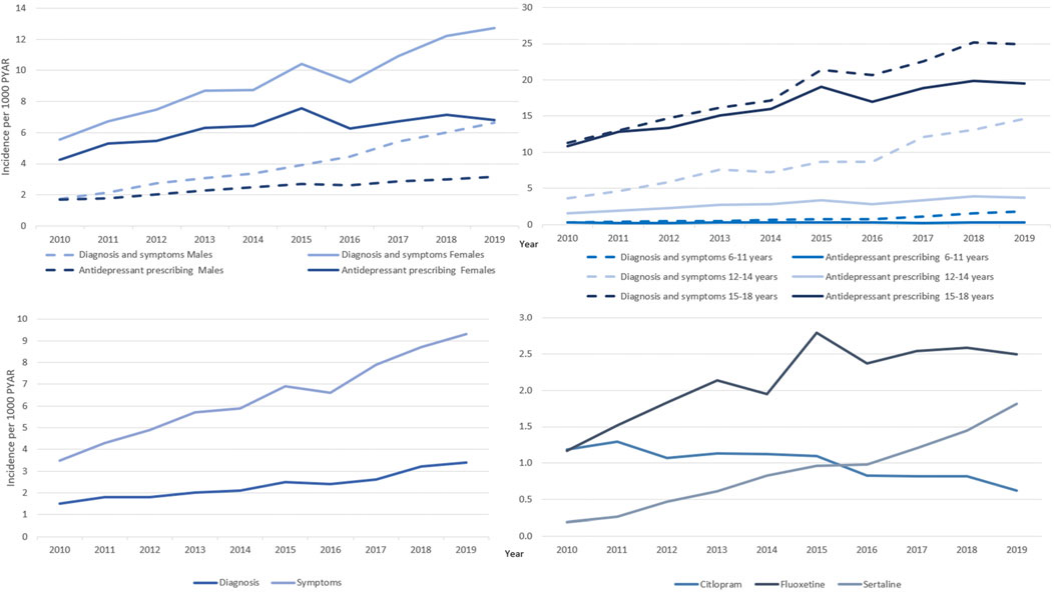
Incidence of depression and antidepressant prescribing over time stratified by sex, age group, symptoms, diagnoses and antidepressant type.

Incidence of depression has more than doubled during the study period rising from 4.6 cases per 1000 Person Years At Risk (PYAR) in 2010 to 12.1 cases per 1000 PYAR in 2019 (IRR 2.8 [95% CI 2.5–3.2]; Table 1). While depression is more commonly recorded in females, the steepest increase over time is seen in males with an almost four-fold increase (males IRR 3.8 [95% CI 3.5–4.4]; females (IRR 2.4 [95 CI 2.1–2.8]; Figure 1).

Incidence of depression increased across all age groups with the largest increase seen in the 6–11-year-old group although incidence remained lower than in older age groups. Incidence increased nearly four-fold in 12–14-year-olds and more than doubled in 15–17-year-olds (IRR 6–11-year-olds 5.3 [95% CI 4.3–6.5]; 12–14-year-olds 3.8 [95% CI 3.3–4.3]; 15–17-year-olds 2.3 [95% CI 2.0–2.6]; Figure 1).

Increases in 6–11 year olds were similar for males and females, with larger increases seen for males in both those aged 12–14 years and those aged 15–17 years (IRRs males 6–11 years 5.4 [95% CI 4.3–6.8]; females 6–11 years 5.2 [95% CI 3.8–6.9]; males 12–14 years 4.3 [95% CI 3.8–4.8]; females 12–14 years 3.6 [95% CI 3.1–4.2]; males 15–17 years 3.6 [95% CI 3.2–4.0]; females 15–17 years 1.9 [95% CI 1.8–2.1]).

When exploring incidence symptoms and diagnoses of depression separately, incidence of symptoms is around 3 times higher than incidence of diagnoses with an increase seen in both over the study period (IRRs diagnoses 2.4 [95% CI 2.1–2.8]; symptoms 2.9 [95% CI 2.5–3.4]; Figure 1 (online Supplementary Table S2)).

### Antidepressant medications

A total of 13 298 (2.1%) individuals were prescribed 13 883 incident antidepressant prescriptions. SSRIs were the most frequently prescribed antidepressant overall. Fluoxetine was the most popular SSRI, with more individuals treated with Fluoxetine than with Citalopram and Sertraline combined. We found very few prescriptions (<50) Paroxetine and Venlafaxine. Prescriptions of duloxetine were also rare (<50) as were prescriptions for Escitalopram (<50). There were more than 500 prescriptions Mirtazapine.

Fluoxetine was the most popular incident prescription accounting for almost half of incident prescriptions, followed by Citalopram, TCAs and Sertraline (Fluoxetine 46.0% [95% CI 44.9–47.1]; Citalopram 20.0% [19.3 to −20.7]; TCAs 16.1% [95% CI 15.5–16.8]; Sertraline 15.2% [95% CI 14.5–15.8]). ‘Other antidepressants’ were the most popular subsequent prescription (75.3% [95% CI 72.7–77.9; *n* = 3251]; online Supplementary Table S3).

#### Indications

Indications for antidepressant prescriptions by antidepressant type, sex and age group are shown in Table 2. Half of antidepressant prescriptions were associated with depression and two thirds with either depression or anxiety. We were able to identify the indications for 59.2% (95% CI 57.2–61.3; *n* = 3117) of the remaining prescriptions. The most common indications after depression and anxiety were pain, including headaches/migraine, followed by other indications of interest, namely attention deficit hyperactivity disorder, conduct disorder, autism, psychosis, stress, and eating disorders, sleep problems and irritable bowel syndrome combined. Enuresis accounted for <1%. No indication was found for 15.5% (95% CI 14.9–16.2; *n* = 2147) of prescriptions.

**Table 2.** *n*, % (95% CI) of antidepressant prescriptions by potential indication for prescription, stratified by antidepressant type, sex and age

		Depression		Depression/Anxiety		Pain		Enuresis		Other		Unknown	
		*n*	% (95% CI)	*n*	% (95% CI)	*n*	% (95% CI)	*n*	% (95% CI)	*n*	% (95% CI)	*n*	% (95% CI)
All antidepressants													
Sex	Male	1757	43.0 (41.0–45.1)	2309	56.5 (54.2–58.9)	1148	28.1 (26.5–29.8)	62	1.5 (1.2–1.9)	1580	38.7 (36.8–40.6)	825	20.2 (18.8–21.6)
	Female	5165	53.0 (51.6–54.5)	6260	64. 2 (62.6–65.8)	4397	45.1 (43.8–46.5)	36	0.4 (0.3–0.5)	3641	37.4 (36.2–38.6)	1322	13.6 (12.8–14.3)
Age group	6–11 years	44	9.0 (6.5–12.1)	110	22.4 (18.5–27.1)	145	29.6 (25.0–34.8)	51	10.4 (7.7–13.7)	198	40.4 (35.0–46.4)	135	27.6 (23.1–32.6)
	12–14 years	866	37.9 (35.5–40.6)	1133	49.6 (46.8–52.6)	889	39.0 (36.4–41.6)	18	0.8 (0.5–1.2)	901	39.5 (36.9–42.1)	463	20.3 (18.5–22.2)
	15–17 years	6012	54.4 (53.0–55.7)	7326	66.2 (64.7–67.8)	4511	40.8 (39.6–42.0)	29	0.3 (0.2–0.4)	4122	37.3 (36.1–38.4)	1549	14.0 (13.3–14.7)
**Total**		**6922**	**50.0 (48.9–51.2)**	**8569**	**61.9 (60.6–63.3)**	**5545**	**40.1 (39.0–41.2)**	**98**	**0.7 (0.6–0.9)**	**5221**	**37.7 (36.7–38.8)**	**2147**	**15.5 (14.9–16.2)**
SSRI													
Sex	Male	1657	48.6 (48.6–48.7)	2168	63.7 (63.6–63.7)	246	7.2 (7.2–7.2)	15	0.4 (0.4–0.5)	387	11.4 (11.4–11.4)	670	19.7 (19.7–19.7)
	Female	4891	62.1 (62.1–62.1)	5877	74.6 (74.6–74.7)	674	8.6 (8.6–8.6)	12	0.2 (0.1–0.2)	421	5.3 (5.3–5.4)	1046	13.3 (13.3–13.3)
Age group	6–11 years	37	12.0 (11.9–12.2)	102	33.0 (32.9–33.2)	50	16.2 (16.1–16.4)	8	2.6 (2.5–2.8)	92	29.8 (29.7–30.0)	77	24.9 (24.8–25.1)
	12–14 years	832	45.7 (45.6–45.7)	1078	59.2 (59.1–59.2)	204	11.2 (11.2–11.2)	<>	<1%	240	13.2 (13.2–13.2)	370	20.3 (20.3–20.3)
	15–17 years	5679	62.1 (62.1–62.1)	6865	75.0 (75.0–75.1)	666	7.3 (7.3–7.3)	<>	<1%	476	5.2 (5.2–5.2)	1269	13.9 (13.9–13.9)
**Total**		**6548**	**58.1 (56.7–59.5)**	**8045**	**71.3 (69.8–72.9)**	**920**	**8.2 (7.6–8.7)**	**27**	**0.2 (0.2–0.3)**	**808**	**7.2 (6.7–7.7)**	**1716**	**15.2 (14.5–16.0)**
TCA													
Sex	Male	40	7.3 (7.3–7.4)	65	11.9 (11.8–12.0)	286	52.4 (52.3–52.5)	46	8.4 (8.4–8.5)	62	11.4 (11.3–11.5)	124	22.7 (22.6–22.8)
	Female	174	10.3 (10.3–10.4)	262	15.6 (15.5–15.6)	1093	64.9 (64.9–64.9)	24	1.4 (1.4–1.5)	179	10.6 (10.6–10.7)	250	14.8 (14.8–14.9)
Age group	6–11 years	<>	<5%	<>	<5%	69	39.7 (39.5–40.0)	42	24.1 (23.9–24.5)	21	12.1 (11.9–12.5)	56	32.2 (32.0–32.6)
	12–14 years	<>	<10%	<>	<10%	286	64.7 (64.6–64.9)	13	2.9 (2.9–3.1)	44	10.0 (9.9–10.1)	90	20.4 (20.3–20.5)
	15–17 years	183	11.3 (11.3–11.4)	279	17.3 (17.3–17.3)	1024	63.4 (63.4–63.5)	15	0.9 (0.9–1.0)	176	10.9 (10.9–10.9)	228	14.1 (14.1–14.2)
**Total**		**214**	**9.6 (8.4–11.0)**	**327**	**14.7 (13.1–16.3)**	**1379**	**61.8 (58.6–65.2)**	**70**	**3.1 (2.4–4.0)**	**241**	**10.8 (9.5–12.3)**	**374**	**16.8 (15.1–18.6)**
Other antidepressants^a^^,^^b^													
Sex	Male	62	45.3 (45.0–45.7)	78	56.9 (56.7–57.4)							32	23.4 (23.1–23.8)
	Female	103	52.8 (52.6–53.2)	124	63.6 (63.4–63.9)							28	14.4 (14.2–14.7)
**Total**		**165**	**49.7 (42.4–57.9)**	**202**	**60.8 (52.7–69.8)**	**51**	**15.4 (11.4–20.2)**	**<5**	**<1%**	**<>**	**<>**	**60**	**18.1 (13.8–23.3)**

<> Numbers masked to prevent disclosure.

a No age breakdown presented due to small numbers.

b No data presented for pain, enuresis and other due to small numbers.

When prescriptions were stratified by antidepressant type, depression/anxiety were the most common indications for both SSRI and Other antidepressants accounting for 71.3% (95% CI 69.8–72.9) and 60.8% (95% CI 52.7–69.8) respectively. For TCAs prescriptions for depression/anxiety were less common (14.7% [95% CI 13.1–16.3]) with comparatively more prescriptions for pain (61.8% [95% CI 58.6–65.2]). TCAs were also more commonly prescribed for enuresis however the percentage of prescriptions remained low (SSRIs <1%; TCA 3.1% ([95% CI 2.4–4.0]; Other <1%]). Other indications of interest were also most common for TCAs.

A higher proportion of females than males had prescriptions associated with depression/anxiety or pain, whereas a higher proportion of males than females had prescriptions associated with other or unknown indications. This was true across antidepressant subtypes.

A smaller proportion of children aged 6–11 years had a prescription associated with depression/anxiety than older age groups. This differed by subtype with depression/anxiety the most common indication for SSRI prescriptions in children aged 6–11 years (33.0% [95% CI 32.9–33.2; *n* = 102]) and pain the most common for TCAs (39.7% [95% CI 39.5–40.0; *n* = 69]) followed by unknown indications and enuresis. Prescriptions for enuresis made up <3% of prescriptions for TCAs for older age groups and <3% of SSRI prescriptions across all age groups.

#### Healthcare settings

The majority (approximately half) of the CYP with a new SSRI prescription were present in primary care only (47.7%, 95% CI 46.5–49.0), and a third (36.6%, 95% CI 35.5%-37.8%) in outpatient psychiatric or pediatric secondary care settings around the time of the prescription. Incident Citalopram prescriptions were less likely to be associated with secondary care settings than Fluoxetine or Sertraline (Primary care only: Citalopram: 64.2% [95%, CI 61.4–67.0; *n* = 2028], Fluoxetine: 40.3% [95% CI 38.8–41.8; *n* = 2703], Sertraline: 41.5% [95% CI 39.1–44.0]; *n* = 1151]); online Supplementary Table S4).

#### Demographic variables

Females were more than twice as likely to have an antidepressant prescription with this difference largest for TCA's (IRR 3.3 [95% CI 3.0–3.6]). However, overall levels of TCA prescriptions remained low for both sexes. Incidence of Fluoxetine and Sertraline was more than twice as high and Citalopram more than three times as high in females than in males (IRRs Citalopram 3.1 [95% CI 2.9–3.4]; Fluoxetine 2.5 [95% CI 2.4–27]); Sertraline 2.2 [95% CI 2.0–2.4]; Table 3).

**Table 3.** Incidence per 1000 PYAR (95% CI) and IRR^a^ (95% CI)^b^ of antidepressant prescriptions in primary care stratified by subtype

	Citalopram		Fluoxetine		Sertraline	
	Incidence	IRR	Incidence	IRR	Incidence	IRR
Year						
2010	4.6 (4.4–4.9)	Reference *p* < 0.001	4.6 (4.4–4.9)	Reference *p* < 0.001	0.2 (0.1–0.2)	Reference *p* < 0.001
2011	5.6 (5.3–5.8)	1.2 (1.0–1.4)	5.6 (5.3–5.8)	1.2 (1.0–1.3)	0.3 (0.2–0.3)	1.4 (1.0–1.9)
2012	6.1 (5.9–6.4)	1.3 (1.2–1.5)	6.1 (5.9–6.4)	1.2 (1.1–1.4)	0.5 (0.4–0.6)	2.4 (1.8–3.3)
2013	7.1 (6.8–7.4)	1.5 (1.3–1.7)	7.1 (6.8–7.4)	1.4 (1.3–1.6)	0.6 (0.5–0.7)	3.2 (2.3–4.4)
2014	7.4 (7.1–7.7)	1.6 (1.4–1.8)	7.4 (7.1–7.7)	1.5 (1.3–1.7)	0.8 (0.7–0.9)	4.4 (3.2–5.9)
2015	8.7 (8.4–9.1)	2.0 (1.7–2.2)	8.7 (8.4–9.1)	1.8 (1.6–2.0)	1.0 (0.9–1.1)	5.2 (3.8–7.3)
2016	8.4 (8.1–8.7)	1.9 (1.7–2.2)	8.4 (8.1–8.7)	1.6 (1.4–1.8)	1.0 (0.9–1.1)	5.4 (4.0–7.3)
2017	10.0 (9.6–10.3)	2.3 (2.1–2.6)	10.0 (9.6–10.3)	1.7 (1.6–1.9)	1.2 (1.1–1.3)	6.8 (5.0–9.3)
2018	11.4 (11.1–11.8)	2.7 (2.4–3.0)	11.4 (11.1–11.8)	1.9 (1.7–2.1)	1.4 (1.3–1.6)	8.4 (6.3–11.3)
2019	12.1 (11.7–12.5)	2.8 (2.5–3.2)	12.1 (11.7–12.5)	1.8 (1.6–2.0)	1.8 (1.7–2.0)	10.3 (7.7–13.8)
Sex						
Male	4.9 (4.8–5.0)	Reference *p* < 0.001	4.9 (4.8–5.0)	Reference *p* < 0.001	0.6 (0.5–0.6)	Reference *p* < 0.001
Female	11.7 (11.5–11.8)	2.4 (2.3–2.5)	11.7 (11.5–11.8)	2.5 (2.4–2.7)	1.2 (1.2–1.3)	2.2 (2.0–2.4)
Age group						
6–11 years	1.0 (0.9–1.0)	Reference *p* < 0.001	1.0 (0.9–1.0)	Reference *p* < 0.001	0.1 (0.0–0.1)	Reference *p* < 0.001
12–14 years	10.0 (9.8–10.2)	10.8 (9.7–12.1)	10.0 (9.8–10.2)	10.2 (8.5–12.2)	0.6 (0.5–0.6)	10.2 (7.9–13.1)
15–17 years	24.2 (23.9–24.6)	26.4 (23.6–29.6)	24.2 (23.9–24.6)	58.1 (48.8–69.3)	3.3 (3.2–3.5)	60.6 (47.6–77.2)
Deprivation						
Least deprived	6.9 (6.7–7.1)	Reference *p* < 0.001	6.9 (6.7–7.1)	Reference *p* < 0.001	0.7 (0.7–0.8)	Reference *p* < 0.001
2	7.3 (7.1–7.6)	1.1 (1.0–1.2)	7.3 (7.1–7.6)	1.0 (1.0–1.1)	0.8 (0.7–0.9)	1.1 (0.9–1.2)
3	7.9 (7.7–8.2)	1.2 (1.1–1.3)	7.9 (7.7–8.2)	1.1 (1.0–1.2)	0.9 (0.8–0.9)	1.2 (1.0–1.4)
4	8.5 (8.3–8.8)	1.3 (1.2–1.4)	8.5 (8.3–8.8)	1.2 (1.2–1.3)	1.0 (0.9–1.0)	1.3 (1.2–1.5)
Most deprived	9.7 (9.5–9.9)	1.5 (1.4–1.6)	9.7 (9.5–9.9)	1.4 (1.3–1.5)	1.0 (1.0–1.1)	1.5 (1.3–1.7)

a Adjusted for calendar year, age, sex and deprivation.

b Based on Wald test.

Rates of all prescriptions increased with age, with the highest rates of prescriptions in the oldest age groups. The Incidence of antidepressant prescriptions was higher in the most deprived compared with the least deprived areas (Tables 1 and 3; Prevalence online Supplementary Table S1).

Fluoxetine made up almost half of first prescriptions for both males and females Sertraline made up a higher proportion of first prescriptions for males than for females with Citalopram and TCAs making up a slightly larger proportion of first prescriptions for females (Fluoxetine, males 47.3% [95% CI 45.2–49.4; *n* = 1932], females 45.4 [95% CI 44.1–46.8; *n* = 4429]; Sertraline, males 18.2% [95% CI 16.9–19.5; *n* = 743], females 13.9 [95% CI 13.2–14.6; 1353]; Citalopram, males 17.4% [95% CI 16.2–18.8; *n* = 712], females 21.1 [95% CI 20.2–22.0; *n* = 2053]; TCAs, males 13.4% [95% CI 12.3–14.5; *n* = 546], females 17.3% [95% CI 16.5–18.1; *n* = 1684] online Supplementary Table S5).

Fluoxetine was the most popular first incident prescription in 6–11-year-olds (38.4% [95% CI 33.1–44.3; *n* = 188]) followed by TCAs (35% [95% CI 30.4–31.2; *n* = 174) and Sertraline (19.0% [95% CI 15.3–23.3; *n*-93]) with Citalopram making up less than 5% first prescriptions for this age group. In those aged 15–17 years Fluoxetine remained the most popular first prescription, followed by Citalopram, with similar proportions of Sertraline and TCAs (Fluoxetine 44.5% [95% CI 43.3–45.8; *n* = 4923]; Citalopram 22.9% [95% CI 22.0–23.8; 95% CI 2533]; Sertraline 14.9% [95% CI 14.2–15.6; *n* = 1648; TCAs 14.6% [95% CI 13.9–15.3; *n* = 1614]).

#### Trends over time

Antidepressant prescribing has increased over the study period, with the steepest increase seen to 2015 (IRR 1.8 [95% CI 1.6–2.0]; Table 1). This has been driven by an increase in prescribing of SSRIs which have doubled from 2.3 (95% CI 2.2–2.5) to 4.4 (95% CI 4.1–4.6) cases per 1000 PYAR over the study period (IRR = 2.0 [95% CI 1.7–2.4; Table 3]). Prescribing of TCAs increased up to 2015 but has decreased in recent years resulting in no significant change overall (2019 IRR 1.1 [95% CI 0.9–1.3]).

Citalopram prescriptions have decreased by around half from 1.2 (95% CI 1.1–1.3) to 0.6 (95% CI 0.5–0.7) cases per 1000 PYAR (IRR 0.6 [95% CI 0.5–0.7]). Fluoxetine prescriptions have more than doubled over time from 1.1 (95% CI 1.1–1.3) to 2.5 (95% CI 2.3–2.7; IRR 2.3 [95% CI 2.0–2.7]). The largest increase over time was seen for Sertraline with a ten-fold increase from 0.2 (95% CI 0.1–0.2) to 1.8 (95% CI 1.7–2.0; IRR 10.3 [95% CI 7.7–13.8]; Figure 1).

### Time series modelling

**Figure 2.**
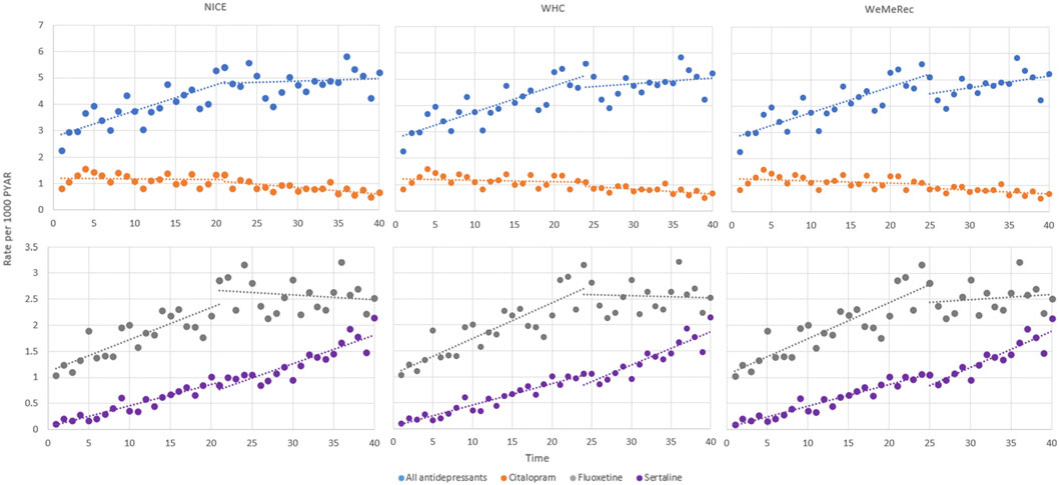
Change in trend of incident antidepressant prescriptions following clinical guidance.

Segmented regression analysis was performed with the interruptions set at time of publication of each of the three guidelines (NICE [March 2015], WHC [October 2015], WeMeRec [March 2016]; Table 4, Fig. 2).

**Table 4.** Time trend analysis of changes in the incidence per 1000 PYAR (95% CI) of antidepressant prescriptions following NICE, WHC, WeMeRec clinical guidelines

	NICE			WHC			WeMeRec	
	Coefficient (95% CLs)	*p*		Coefficient (95% CLs)	*p*		Coefficient (95% CLs)	*p*
All antidepressants								
Pre slope	0.09 (0.05–0.13)	<0.001	Pre slope	0.09 (0.06–0.12)	<0.001	Pre slope	0.10 (0.07–0.13)	<0.001
Post slope	0.01 (−0.03 to 0.05)	0.629	Post slope	0.02 (−0.03 to 0.07)	0.360	Post slope	0.04 (−0.01 to 0.10)	0.081
Change in level	0.05 (−0.57 to 0.67)	0.873	Change in level	−0.37 (−0.99 to 0.24)	0.226	Change in level	−0.76 (−1.35 to −0.17)	0.013
Change in slope	−0.08 (−0.14 to −0.03)	0.004	Change in slope	−0.07 (−0.13 to −0.02)	0.015	Change in slope	−0.06 (−0.11 to 0.00)	0.061
Fluoxetine								
Pre slope	0.05 (0.03–0.08)	<0.001	Pre slope	0.06 (0.04–0.08)	<0.001	Pre slope	0.07 (0.05–0.09)	<0.001
Post slope	−0.01 (−0.03 to 0.01)	0.438	Post slope	−0.00 (−0.03 to 0.03)	0.776	Post slope	0.01 (−0.02 to 0.04)	0.533
Change in level	0.37 (−0.00 to 0.73)	0.052	Change in level	−0.03 (−0.41 to 0.4)	0.894	Change in level	−0.33 (−0.70 to 0.05)	0.089
Change in slope	−0.06 (−0.09 to −0.03)	<0.001	Change in slope	−0.07 (−0.10 to −0.03)	<0.001	Change in slope	−0.06 (−0.10 to −0.02)	0.002
Citalopram								
Pre slope	−0.01 (−0.02 to 0.01)	0.428	Pre slope	−0.01 (−0.02 to 0.01)	0.316	Pre slope	−0.01 (−0.02 to 0.01)	0.287
Post slope	−0.02 (−0.04 to −0.01)	0.003	Post slope	−0.02 (−0.04 to 0.00)	0.052	Post slope	−0.02 (−0.04 to 0.01)	0.145
Change in level	−0.04 (−0.29 to 0.21)	0.745	Change in level	−0.14 (−0.38 to 0.12)	0.254	Change in level	−0.19 (−0.43 to 0.05)	0.119
Change in slope	−0.02 (−0.04 to 0.00)	0.100	Change in slope	−0.01 (−0.04 to 0.01)	0.257	Change in slope	−0.01 (−0.03 to 0.01)	0.433
Sertraline								
Pre slope	0.04 (0.03–0.05)	<0.001	Pre slope	0.04 (0.03–0.05)	<0.001	Pre slope	0.04 (0.03–0.05)	<0.001
Post slope	0.06 (0.04–0.07)	<0.001	Post slope	0.06 (0.05–0.08)	<0.001	Post slope	0.06 (0.05–0.08)	<0.001
Change in level	−0.15 (−0.33 to 0.03)	0.104	Change in level	−0.19 (−0.36 to −0.02)	0.029	Change in level	−0.19 (−0.36 to −0.02)	0.029
Change in slope	0.01 (−0.00 to 0.03)	0.089	Change in slope	0.02 (0.01–0.04)	0.006	Change in slope	0.02 (0.01–0.04)	0.006

Incidence of all antidepressants, Fluoxetine and Sertraline were increasing significantly, and Citalopram was decreasing non-significantly prior to the issuance of guidance. Following guidance there was a no significant step-change but there was a significant change in trend for all antidepressants with the increase over time no longer reaching statistical significance (all antidepressants change in slope after NICE guidance −0.08 [95% CLs 0.14–0.03; *p* = 0.004]; WHC −0.07 [95% CLs −0.13 to −0.02; *p* = 0.015]; WeMeRec −0.06 [95% CLs −0.11–0.00; *p* = 0.061]).

There was both a significant step-change decrease and a significant change in trend for Fluoxetine with a flattening of the slope following guidance (Fluoxetine change in slope after NICE guidance −0.06 [95% CLs −0.09 to –0.03; *p* < 0.001; WHC −0.07 [95% CLs −0.10 to −0.03; *p* < 0.001]; WeMeRec −0.06 [95% CLs −0.10 to −0.02; *p* < 0.001]).

No significant step-change or change in trend was seen for rates of Citalopram prescribing with a non-significant decrease in prescriptions over time seen both before and after guidance was issued.

There was no significant step-change in level of Sertraline but there was a significant change in trend following WHC and WeMeRec guidance with the upward trend in prescriptions becoming steeper after guidance was issued (change in slope after NICE guidance 0.01 [95% CLs 0.00–0.03; *p* = 0.089; WHC 0.02 [95% CLs 0.01–0.04; *p* = 0.006; WeMeRec 0.02 [95% CLs 0.01–0.04; *p* = 0.006]).

## Discussion

### Summary of findings

This study set out to explore recent trends in depression and antidepressant prescribing in CYP and to evaluate whether national guidelines had any impact on prescribing choices. Incidence of recorded depression has more than doubled over the study period with the steepest increase in males. Incidence of both depression and antidepressant prescribing was highest in the most deprived compared with least deprived quintiles. There were associations between guidance being issued and subsequent changes in prescribing behavior with the previously upward trend in prescribing of Fluoxetine levelling off to become non-significant. In contrast the upward trend in Sertraline prescriptions increased further. Citalopram prescriptions have decreased overall during the study period with no significant change following guidance.

### Comparison with previous literature

#### Depression

Rates of depression have continued to rise in keeping with previous research, with a continued preference for recording of symptoms over diagnoses (John et al., 2016b). This study additionally demonstrates that a record of a diagnosis is more likely to be treated with antidepressants than a record of symptoms. Depression and prescribing were more common in adolescent girls and those living in the most deprived areas. High levels of depression in adolescent girls is consistent across community and clinical samples, suggesting a genuine increasing trend (Ford, John, & Gunnell, 2021; Thapar et al., 2012, 2022). Levels of emotional distress (particularly anxiety) and a reduction in emotional wellbeing in girls have been reported since 2011 (Fink et al., 2015; NHS Digital, 2018; 2022; Pitchforth et al., 2019; Sarginson et al., 2017). This may be attributable to modern societal pressures including chronic adversity, stressors from negative relationships, rising levels of poverty, increasing academic pressures, and increased use of social media (Chandan et al., 2020; Degli Esposti et al., 2019; Deighton et al., 2019; Restifo & Bogels, 2009).

While levels of depression were lower in boys an almost four-fold increase in incidence of depression was found in this group. Research with CPRD data in England has also found a larger increase in depression in males than females aged 13–19 from 2003–2018 (Cybulski et al., 2021) further supported by routine data in adolescents in Germany (Steffen, Thom, Jacobi, Holstiege, & Bätzing, 2020). This differs from survey or clinical interview data which suggests a larger increase in females over time (e.g. (Daly, 2022; Keyes, Gary, O'Malley, Hamilton, & Schulenberg, 2019; Platt, Bates, Jager, McLaughlin, & Keyes, 2021)). A recent review of studies concluded that increases in depression were greatest in adolescent females based on survey data with an increasing gender gap over time for adolescents but not for adults (Thapar et al., 2022). The increase seen in routine data may be partly attributable to increases in help-seeking, recognition, and treatment of depression in males. This may also be related to increasing rates of diagnosed ADHD and ASD over time, which are more common in males and are associated with high rates of early-onset depression (Thapar, Livingston, Eyre, & Riglin, 2023). Further research is needed to explore this increase and potential avenues for intervention and support.

#### Antidepressant prescriptions

Fluoxetine is the recommended first line treatment for depression based on efficacy and safety and is recommended for use in combination with psychotherapy (Goodyer et al., 2007, 2008; Hetrick et al., 2007; March et al., 2004). If Fluoxetine has been unsuccessful, Sertraline and Citalopram are recommended as the second line treatments (Hopkins, Crosland, Elliott, & Bewley, 2015; NICE, 2019; Sakolsky & Birmaher, 2012). This study demonstrates only partial adherence to this guidance.

Fluoxetine was the most popular antidepressant overall. In keeping with previous UK research, around half of incident antidepressant prescriptions were associated with depression with other common indications including pain and anxiety (John et al., 2016a; Sarginson et al., 2017). TCAs made up a larger proportion of incident prescriptions for 6–11-year-olds than for older age groups. We found that TCAs were mostly commonly associated with pain, followed by other indications and enuresis in this age group rather than for depression or anxiety in keeping with previous research (Murray, de Vries, & Wong, 2004; Sarginson et al., 2017).

No recorded indication was found for 22% of cases in the current study compared with 17% of cases in previous research (John et al., 2016b). We also explored whether prescribing was initiated in secondary care as recommended. Around half of new prescriptions were associated with primary care contact only. While this analysis is limited by the data (see below) an absence of secondary care contact is in keeping with previous studies (Dai Cao et al., 2021). Prescribing in primary care may be related to access to specialist mental health services and long waiting lists as demand and the rising incidence of mental health problems in CYP far outstrip capacity of specialist mental health services across many countries leaving limited scope for referring to secondary care and few alternative treatment options (Hetrick, McKenzie, Cox, Simmons, & Merry, 2012; Hyde et al., 2005).

Citalopram prescribing was less likely to be associated with secondary care settings and was considerably higher for females compared to Sertraline and Fluoxetine. Fluoxetine was the most popular first-line antidepressant across age groups. However, Citalopram was prescribed in the first instance more often in older age groups (<5% of first prescriptions in 6–11-year-olds compared with around a fifth in 15–17-year-olds). The reasons why GPs may preferentially prescribe Citalopram to treat adolescent girls is unclear but could reflect prescribing preferences and familiarity for adult patients.

The current study further extends previous work (John et al., 2016a) demonstrating a continued increase in rates of Fluoxetine prescriptions that more than doubled between 2010 and 2015, followed by a levelling of the slope following the 2015/2016 guidance. Rates of Citalopram prescriptions have continued to decline. Sertraline prescriptions increased more than ten-fold over time supported by previous research (Dai Cao et al., 2021; Jack et al., 2020) with a significant step change and an increase in slope for Sertraline prescriptions following the 2015/2016 guidance. A change in prescribing preference from Citalopram to Sertraline may be based on an understanding that Sertraline has fewer side effects and greater efficacy (Johnson, Williams, MacGillivray, Dougall, & Maxwell, 2017; Wagner et al., 2002). Sertraline may also be considered more suitable for mixed anxiety and depression than Citalopram or Fluoxetine (Garland, Kutcher, Virani, & Elbe, 2016; Hetrick et al., 2012). Increases in Sertraline prescriptions may also be attributable to being mentioned in the guidance even though cautions for its use were clearly given (Tiffin et al., 2019).

### Strengths and limitations

This study, derived from a large representative population of CYP studied over 9 years, provides a comprehensive picture on which to base targeted intervention, resources, and service provision. This study utilized population level data from Wales and findings while generalizable to the rest of the UK, may not be generalizable internationally. Of note we explored changes in prescribing following three different sets of guidance, one at national level (NICE) and two for Wales only (WHC, WeMeRec). Further research could confirm whether the effect of the national guidance was mirrored across the UK, however prescribing patterns are consistent with those found in routinely collected healthcare data in England (Dai Cao et al., 2021; Jack et al., 2020).

Routinely collected data have limitations for research purposes, and the quality and completeness of data varies across datasets. We have attempted to minimize the impact of this by only including GPs that meet standards for data quality and using validated code lists (John et al., 2016b). Data completeness with outpatients' data varies across health boards and over time and as such results should be interpreted with caution. Depression not resulting in presentation to services, or where depression is discussed but not recorded will not be captured here. This is a common feature of all studies using routine data. These data are a reflection of contacts with the healthcare system and not rates of depression in the community.

Prescribing data is well reported in primary care; however, at present, it is not possible to assess if medications were dispensed or taken. Future work could look to build on analysis here exploring repeat prescriptions as a possible marker of adherence to treatment. Further work, including qualitative, is also required to better understand the increase in depression presenting to healthcare settings in young males. There is some evidence that individuals living in more affluent areas are more likely to access private psychotherapy (Jokela, Batty, Vahtera, Elovainio, & Kivimäki, 2013). It is not possible based on the current data to assess access to private care.

While we explored a more extensive list of indications for which an antidepressant may be prescribed than in some previous research (Murray et al., 2004) prescriptions are not explicitly linked to the condition for which the medication has been prescribed and may not even be recorded within GP data. Indications can only be inferred for those patients with relevant codes in the patient records related to defined times around prescription and are reflective of coding behavior by clinicians. Diagnoses such as depression, especially if chronic or recurrent may not be regularly recorded. As such ascertaining indications based on temporal proximity is likely to have underestimated depression as an indication. In addition, diagnoses made where the individual was not registered with a SAIL supplying practice will not be captured. We have extended previous research exploring indications in primary care only (John et al., 2016a) to explore whether prescriptions may have been initiated in secondary care. To do this we have assumed that if a patient attended secondary care within the previous year for a mental health condition that the source of the prescribing has originated from there. This method would result in an under-estimation of prescribing initiated by GPs.

### Implications

Rates of depression are increasing in CYP, and the steep increase in depression amongst adolescent males is in keeping with other studies utilizing healthcare data (Cybulski et al., 2021; Steffen et al., 2020) but differs from some research utilizing community samples (Thapar et al., 2022). This potentially suggests an increase in help seeking, recognizing or treatment of depression in young males in primary care. This may also be related to increases in ADHD and ASD diagnoses in recent years and their association with early-onset depression (Thapar et al., 2023). Primary Care is an important point of contact. It is important that GPs are supported in their ability to identify CYP with depression, and to determine when referral to specialist services is necessary. Additional training may be needed for prescribers including depression recognition, suicide risk, and communication techniques for managing the wider social aspects that may have an influence over prescribing choices. Future work could explore contacts in more detail, for example looking at wider range of possible indications for antidepressant prescriptions accounting for clinical history across healthcare settings, as well as co-prescription of other medications. Further exploration of self-harm and suicide rates in CYP who have been prescribed antidepressants could better inform guidance.

Practitioners are partially adhering to local and national guidance. The decision-making behind prescribing choices is likely to be multi-factorial, based upon patient, family socio-economic and drug-specific factors as well as prescribing guidelines, the media, and historical prescribing practice. Previous research has found that decisions and treatment options are severely constrained by lack of time and inadequate access to specialist services (Hyde et al., 2005; Pollock & Grime, 2003; Rogers, 2001). It may be difficult for GPs to implement guidelines if there are long waiting lists for specialist care. This may result in increased pressure to prescribe based on some of the above factors. Strategies for change in provider behavior may need to go beyond guidance (Klein et al., 2001). Guidance in conjunction with further funding for secondary services provision, to ensure timely access for CYP to services would ensure that GPs have the resources needed to support CYP.

Further research is needed to explore trends over time from 2019 onwards and how prescribing behaviors may have changed over the course of the pandemic, with associated strains on specialist mental health settings.

## Conclusions

This study contributes to a growing body of research that depression in CYP has continued to increase. Fluoxetine is currently the most popular incident antidepressant prescription in CYP with incidence of Citalopram prescriptions continuing to decrease. In contrast to guidance focused on the prescription of Fluoxetine as the antidepressant of choice in CYP, rates of Sertraline have increased ten-fold over time with a steepening of this increase following local and national guidance (2015/2016).

Decision making around antidepressant prescriptions is influenced by factors outside of national and local published guidelines including responses to demand, practitioner-patient relationships and whether secondary services can be accessed expeditiously. Further research is needed to better understand factors influencing GP prescribing. New strategies and targeted interventions are urgently needed, including increased and focused investment to address the scale of unmet need by improving specialist service capacity and access to these services.

## Supporting information

Marchant et al. supplementary material Marchant et al. supplementary material

## Supplementary material

The supplementary material for this article can be found at https://doi.org/10.1017/S0033291724002861.
