# Supplementary material for: The impact of published guidance on trends in the pharmacological management of depression in children and adolescents- a whole population e-cohort data linkage study in Wales, UK
Source: Psychol Med. 2025 Jan 8;54(16):4691–703. doi: 10.1017/S0033291724002861 (PMC11779557; doi:10.1017/S0033291724002861)
Supplement: Marchant et al. supplementary material [file S0033291724002861sup001.docx]

**
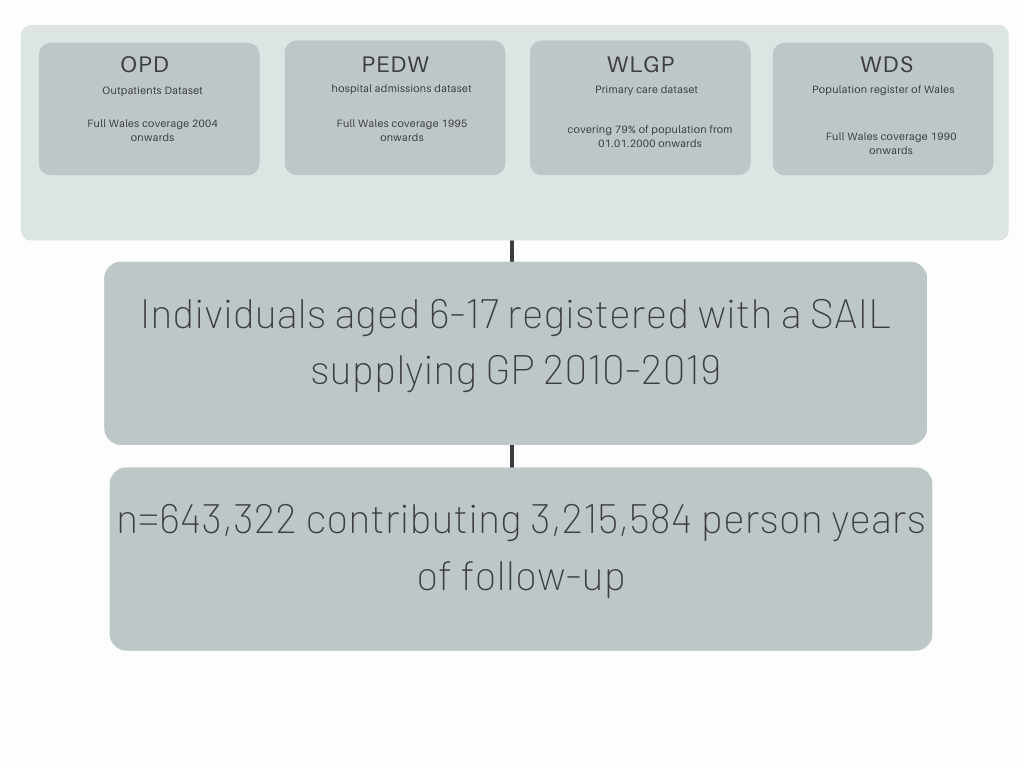
**

Supplementary Figure 1 Cohort Data flow diagram

Supplementary Table 1 Prevalence per 1000 PYAR (95% CI) and IRR^a^ (95% CI)^b^ of depression and antidepressant prescriptions in primary care

|  |  | Depression diagnosis and Symptoms combined | | All Antidepressants | | Citalopram | | Fluoxetine | | Sertraline | |
| --- | --- | --- | --- | --- | --- | --- | --- | --- | --- | --- | --- |
| Year |  | Prevalence | IRR | Prevalence | IRR | Prevalence | IRR | Prevalence | IRR | Prevalence | IRR |
|  | 2010 | 5.0(4.8-5.3) | Reference p<.001 | 5.0(4.8-5.3) | Reference p<.001 | 1.4(1.3-1.5) | Reference p<.001 | 1.7(1.5-1.8) | Reference p<.001 | 0.3(0.2-0.3) | Reference p<.001 |
|  | 2011 | 6.1(5.8-6.4) | 1.2(1.0-1.4) | 6.1(5.8-6.4) | 1.2(1.1-1.4) | 1.6(1.5-1.8) | 1.1(1.0-1.3) | 2.2(2.1-2.4) | 1.3(1.1-1.5) | 0.4(0.3-0.5) | 1.4(1.0-2.0) |
|  | 2012 | 6.8(6.5-7.0) | 1.3(1.2-1.5) | 6.8(6.5-7.0) | 1.4(1.2-1.6) | 1.5(1.3-1.6) | 1.0(0.9-1.2) | 2.7(2.5-2.9) | 1.6(1.4-1.8) | 0.6(0.6-0.7) | 2.5(1.8-3.3) |
|  | 2013 | 7.7(7.4-8.0) | 1.5(1.4-1.7) | 7.7(7.4-8.0) | 1.6(1.4-1.8) | 1.5(1.3-1.6) | 1.0(0.9-1.2) | 3.2(3.0-3.4) | 1.9(1.6-2.2) | 0.9(0.8-1.0) | 3.4(2.6-4.5) |
|  | 2014 | 8.2(7.9-8.5) | 1.7(1.5-1.9) | 8.2(7.9-8.5) | 1.8(1.6-2.0) | 1.5(1.3-1.6) | 1.1(0.9-1.2) | 3.4(3.2-3.6) | 2.0(1.7-2.3) | 1.2(1.1-1.3) | 4.7(3.7-6.1) |
|  | 2015 | 9.6(9.2-9.9) | 2.0(1.8-2.2) | 9.6(9.2-9.9) | 2.0(1.8-2.3) | 1.5(1.3-1.6) | 1.1(0.9-1.2) | 4.2(4.0-4.5) | 2.6(2.3-3.0) | 1.4(1.3-1.6) | 5.7(4.4-7.5) |
|  | 2016 | 9.4(9.1-9.7) | 2.0(1.8-2.2) | 9.4(9.1-9.7) | 2.0(1.8-2.2) | 1.2(1.1-1.3) | 0.9(0.8-1.0) | 4.1(3.9-4.3) | 2.5(2.2-2.9) | 1.6(1.4-1.7) | 6.5(5.0-8.4) |
|  | 2017 | 10.9(10.6-11.3) | 2.3(2.1-2.6) | 10.9(10.6-11.3) | 2.0(1.8-2.3) | 1.1(1.0-1.2) | 0.8(0.7-1.0) | 4.0(3.8-4.3) | 2.6(2.3-2.9) | 1.8(1.7-2.0) | 7.6(5.8-9.9) |
|  | 2018 | 12.6(12.2-12.9) | 2.7(2.4-3.1) | 12.6(12.2-12.9) | 2.2(2.0-2.5) | 1.1(1.0-1.2) | 0.9(0.8-1.0) | 4.3(4.1-4.5) | 2.8(2.4-3.2) | 2.1(2.0-2.3) | 9.2(7.1-11.9) |
|  | 2019 | 13.3(13.0-13.8) | 2.9(2.5-3.3) | 13.3(13.0-13.8) | 2.2(2.0-2.5) | 0.9(0.8-1.0) | 0.7(0.6-0.8) | 4.2(4.0-4.4) | 2.7(2.4-3.1) | 2.6(2.4-2.8) | 10.9(8.5-14.0) |
| Gender | Male | 5.2(5.1-5.3) | Reference p<.001 | 5.2(5.1-5.3) | Reference p<.001 | 0.6(0.6-0.7) | Reference p<.001 | 2.1(2.0-2.2) | Reference p<.001 | 0.9(0.9-1.0) | Reference p<.001 |
|  | Female | 13.0(12.8-13.1) | 2.5(2.4-2.6) | 13.0(12.8-13.1) | 2.4(2.3-2.5) | 2.0(1.9-2.1) | 3.1(2.9-3.4) | 4.8(4.7-4.9) | 2.3(2.2-2.4) | 1.7(1.7-1.8) | 1.9(1.8-2.1) |
| Age group | 6-11 years | 1.0(0.9-1.0) | Reference p<.001 | 1.0(0.9-1.0) | Reference p<.001 | 0.0(0.0-0.0) | Reference p<.001 | 0.2(0.2-0.2) | Reference p<.001 | 0.1(0.1-0.1) | Reference p<.001 |
|  | 12-14 years | 10.6(10.4-10.9) | 11.3(10.0-12.6) | 10.6(10.4-10.9) | 9.5(7.9-11.3) | 0.3(0.3-0.4) | 12.5(9.0-17.6) | 2.4(2.3-2.5) | 13.4(10.6-16.9) | 0.8(0.8-0.9) | 10.3(8.1-13.1) |
|  | 15-17 years | 27.3(26.9-27.7) | 29.1(25.9-32.6) | 27.3(26.9-27.7) | 58.6(49.3-69.6) | 5.6(5.5-5.8) | 202.1(147.1-277.7) | 12.8(12.6-13.1) | 72.9(58.3-91.3) | 4.9(4.7-5.1) | 61.1(48.8-76.6) |
| Deprivation | Least deprived | 7.5(7.3-7.8) | Reference p<.001 | 7.5(7.3-7.8) | Reference p<.001 | 0.9(0.8-1.0) | Reference p<.001 | 3.3(3.1-3.4) | Reference p<.001 | 1.2(1.1-1.3) | Reference p<.001 |
|  | 2 | 8.1(7.8-8.3) | 1.1(1.0-1.2) | 8.1(7.8-8.3) | 1.0(0.9-1.1) | 1.1(1.0-1.2) | 1.2(1.1-1.4) | 3.1(2.9-3.2) | 0.9(0.9-1.0) | 1.2(1.1-1.3) | 1.0(0.9-1.1) |
|  | 3 | 8.7(8.5-8.9) | 1.2(1.1-1.2) | 8.7(8.5-8.9) | 1.1(1.0-1.2) | 1.3(1.3-1.4) | 1.5(1.4-1.7) | 3.3(3.1-3.4) | 1.0(0.9-1.1) | 1.3(1.2-1.4) | 1.1(1.0-1.2) |
|  | 4 | 9.4(9.1-9.6) | 1.3(1.2-1.4) | 9.4(9.1-9.6) | 1.2(1.1-1.3) | 1.4(1.3-1.5) | 1.5(1.4-1.7) | 3.5(3.4-3.7) | 1.1(1.0-1.2) | 1.4(1.3-1.5) | 1.2(1.1-1.3) |
|  | Most deprived | 10.7(10.4-10.9) | 1.5(1.4-1.6) | 10.7(10.4-10.9) | 1.3(1.2-1.4) | 1.6(1.6-1.7) | 1.9(1.7-2.1) | 3.8(3.6-3.9) | 1.2(1.1-1.3) | 1.4(1.3-1.5) | 1.3(1.1-1.4) |
| 1. Adjusted for calendar year, age, sex and deprivation 2. Based on Wald test | | | | | | | | | | | |

Supplementary Table 2 Incidence and Prevalence per 1000 PYAR (95% CI) and IRR^a^ (95% CI)^b^ of depression diagnosis and symptoms in primary care

|  |  | | Depression diagnosis | | | | | Depression symptoms | | |  | |
| --- | --- | --- | --- | --- | --- | --- | --- | --- | --- | --- | --- | --- |
| Year |  | Incidence | | IRR | Prevalence | IRR | Incidence | | IRR | Prevalence | | IRR |
|  | 2010 | 1.5(1.4-1.7) | | Reference p<.001 | 1.6(1.4-1.7) | Reference p<.001 | 3.5(3.3-3.7) | | Reference p<.001 | 3.8(3.6-4.0) | | Reference p<.001 |
|  | 2011 | 1.8(1.7-2.0) | | 1.2(1.0-1.4) | 1.9(1.8-2.1) | 1.2(1.0-1.4) | 4.3(4.1-4.5) | | 1.2(1.0-1.4) | 4.6(4.4-4.9) | | 1.2(1.0-1.4) |
|  | 2012 | 1.8(1.6-1.9) | | 1.1(1.0-1.3) | 1.9(1.7-2.0) | 1.1(1.0-1.3) | 4.9(4.7-5.2) | | 1.4(1.2-1.6) | 5.3(5.1-5.6) | | 1.4(1.2-1.6) |
|  | 2013 | 2.0(1.9-2.2) | | 1.3(1.1-1.5) | 2.1(1.9-2.3) | 1.3(1.1-1.5) | 5.7(5.5-6.0) | | 1.6(1.4-1.9) | 6.1(5.9-6.4) | | 1.6(1.4-1.9) |
|  | 2014 | 2.1(2.0-2.3) | | 1.4(1.2-1.6) | 2.2(2.1-2.4) | 1.4(1.2-1.6) | 5.9(5.6-6.1) | | 1.7(1.5-1.9) | 6.4(6.2-6.7) | | 1.7(1.5-2.0) |
|  | 2015 | 2.5(2.3-2.7) | | 1.7(1.5-1.9) | 2.6(2.4-2.8) | 1.7(1.5-1.9) | 6.9(6.6-7.2) | | 2.0(1.8-2.3) | 7.5(7.2-7.8) | | 2.1(1.8-2.4) |
|  | 2016 | 2.4(2.2-2.5) | | 1.6(1.4-1.9) | 2.5(2.4-2.7) | 1.7(1.4-1.9) | 6.6(6.3-6.9) | | 2.0(1.7-2.3) | 7.2(7.0-7.5) | | 2.0(1.8-2.3) |
|  | 2017 | 2.6(2.4-2.8) | | 1.8(1.6-2.1) | 2.8(2.6-3.0) | 1.9(1.7-2.1) | 7.9(7.6-8.2) | | 2.4(2.1-2.8) | 8.4(8.1-8.8) | | 2.4(2.1-2.8) |
|  | 2018 | 3.2(3.0-3.4) | | 2.3(2.1-2.6) | 3.4(3.2-3.6) | 2.3(2.1-2.6) | 8.7(8.4-9.0) | | 2.7(2.4-3.1) | 9.5(9.2-9.8) | | 2.8(2.4-3.2) |
|  | 2019 | 3.4(3.2-3.6) | | 2.4(2.1-2.8) | 3.6(3.4-3.8) | 2.4(2.1-2.8) | 9.3(8.9-9.6) | | 2.9(2.5-3.4) | 10.0(9.7-10.4) | | 2.9(2.5-3.4) |
| Gender | Male | 1.3(1.2-1.3) | | Reference p<.001 | 1.3(1.2-1.4) | Reference p<.001 | 3.9(3.8-4.0) | | Reference p<.001 | 4.1(4.0-4.2) | | Reference p<.001 |
|  | Female | 3.5(3.4-3.6) | | 2.8(2.7-3.0) | 3.7(3.6-3.8) | 2.9(2.7-3.0) | 9.1(8.9-9.2) | | 2.3(2.2-2.5) | 9.9(9.7-10.0) | | 2.4(2.3-2.5) |
| Age group | 6-11 years | 0.1(0.1-0.1) | | Reference p<.001 | 0.1(0.1-0.1) | Reference p<.001 | 0.8(0.8-0.9) | | Reference p<.001 | 0.9(0.8-0.9) | | Reference p<.001 |
|  | 12-14 years | 2.0(1.9-2.1) | | 22.2(18.2-26.9) | 2.0(1.9-2.1) | 22.5(18.5-27.4) | 8.4(8.2-8.6) | | 10.1(9.0-11.4) | 8.8(8.6-9.0) | | 10.5(9.3-11.8) |
|  | 15-17 years | 8.4(8.2-8.7) | | 94.7(78.3-114.5) | 8.9(8.7-9.2) | 99.6(82.3-120.5) | 18.1(17.8-18.4) | | 22.1(19.7-24.8) | 20.0(19.7-20.3) | | 23.9(21.3-26.8) |
| Deprivation | Least deprived | 2.0(1.8-2.1) | | Reference p<.001 | 2.0(1.9-2.2) | Reference p<.001 | 5.5(5.4-5.7) | | Reference p<.001 | 6.0(5.8-6.2) | | Reference p<.001 |
|  | 2 | 2.0(1.9-2.2) | | 1.0(1.0-1.1) | 2.1(2.0-2.3) | 1.0(1.0-1.1) | 6.0(5.7-6.2) | | 1.1(1.0-1.2) | 6.5(6.3-6.7) | | 1.1(1.0-1.2) |
|  | 3 | 2.2(2.1-2.3) | | 1.1(1.1-1.2) | 2.3(2.2-2.4) | 1.1(1.1-1.2) | 6.4(6.2-6.6) | | 1.2(1.1-1.3) | 6.9(6.7-7.1) | | 1.2(1.1-1.3) |
|  | 4 | 2.5(2.3-2.6) | | 1.3(1.2-1.4) | 2.6(2.4-2.7) | 1.3(1.2-1.4) | 6.9(6.7-7.1) | | 1.3(1.2-1.4) | 7.4(7.2-7.7) | | 1.3(1.2-1.4) |
|  | Most deprived | 2.9(2.8-3.0) | | 1.6(1.5-1.7) | 3.0(2.9-3.2) | 1.6(1.5-1.7) | 7.7(7.5-7.9) | | 1.5(1.3-1.6) | 8.3(8.1-8.5) | | 1.5(1.4-1.6) |
| 1. Adjusted for calendar year, age, sex and deprivation 2. Based on Wald test | | | | | | | | | | | | |

Supplementary Table 3 Prescription number^a^ %(95% CI; n) by antidepressant type

|  | Prescription One | Prescription Two | Prescription Three | Prescription Four+^b^ |
| --- | --- | --- | --- | --- |
| Fluoxetine | 46.2(45.0-47.3; 6139) | 11.7(10.7-12.7; 504) | 5.5(3.9-7.5; 38) | <5 |
| Citalopram | 20.3(19.5-21.0; 2695) | 8.3(7.5-9.2; 360) | 7.9(6.0-10.3; 55) | 9.6(4.4-18.2; 9) |
| Sertraline | 14.8(14.2-15.5; 1968) | 16.0(14.9-17.3; 692) | 9.6(7.5-12.2; 67) | 6.4(2.3-13.9; 6) |
| Other SSRI | 0.5(0.4-0.6; 66) | <5 | <5 | <5 |
| TCA | 16.2(15.5-16.9; 2154) | 2.4(2.0-2.9; 105) | 3.0(1.9-4.6; 21) | <5 |
| Other | 2.3(2.0-2.5; 301) | 61.6(59.2-64.0; 2659) | 74.0(67.7-80.7; 515) | 81.9(64.6-102.4; 77) |
| 1. Prescription number refers to the order in which antidepressants were prescribed as reflection of whether each antidepressant is prescribed as first line, second line etc 2. Refers to prescription number 4 or higher | | | | |
| <> masked to avoid deductive calculation of small numbers in line with SAIL databank data protection policies | | | |  |

Supplementary Table 4 n, %(95% CI) of individuals prescribed incident antidepressant prescriptions linked to each healthcare setting

|  | Primary care | | Outpatients | | Inpatients | | Primary care only | |
| --- | --- | --- | --- | --- | --- | --- | --- | --- |
|  | % | n | % | N | % | n | % | n |
| Antidepressants (n=13298) | 74.9(73.5-76.4) | 9966 | 31.4(30.5-32.4) | 4175 | 7.0(6.6-7.5) | 936 | 45.1(44.0-46.3) | 5997 |
| SSRIs  (n=11075) | 81.2(79.5-82.9) | 8993 | 36.6(35.5-37.8) | 4056 | 7.8(7.3-8.3) | 862 | 47.7(46.5-49.0) | 5287 |
| Fluoxetine  (n=6712) | 81.4(79.2-83.5) | 5461 | 46.2(44.6-47.8) | 3100 | 9.8(9.1-10.6) | 659 | 40.3(38.8-41.8) | 2703 |
| Citalopram  (n=3160) | 81.1(78.0-84.3) | 2563 | 14.2(13.0-15.6) | 450 | 4.7(4.0-5.5) | 149 | 64.2(61.4-67.0) | 2028 |
| Sertraline  (n=2774) | 81.8(78.5-85.3) | 2270 | 45.7(43.2-48.3) | 1267 | 9.2(8.1-10.4) | 254 | 41.5(39.1-44.0) | 1151 |
| TCAs  (n=2294) | 45.3(42.6-48.1) | 1039 | 8.0(6.9-9.2) | 183 | 4.0(3.2-4.9) | 92 | 30.2(28.0-32.5) | 693 |

Supplementary Table 5 Proportion (%[[95% CI; n])^a^ of incident prescriptions for each antidepressant type^b^

|  |  | All incident prescriptions (n) | Fluoxetine | Citalopram | Sertraline | TCA | Other |
| --- | --- | --- | --- | --- | --- | --- | --- |
| Year | 2010 | 933 | 36.5(32.8-40.6; 341) | 36.3(32.6-40.4; 339) | 3.9(2.7-5.3; 36) | 20.6(17.8-23.7; 192) | 2.0(1.2-3.2; 19) |
|  | 2011 | 1115 | 38.6(35.0-42.4; 430) | 32.5(29.2-36.0; 362) | 4.8(3.6-6.3; 54) | 20.7(18.1-23.6; 231) | 2.9(2.0-4.1; 32) |
|  | 2012 | 1171 | 45.8(42.0-49.8; 536) | 25.5(22.7-28.6; 299) | 7.8(6.3-9.5; 91) | 18.3(15.9-20.9; 214) | 2.5(1.7-3.6; 29) |
|  | 2013 | 1342 | 46.5(42.9-50.3; 624) | 23.0(20.5-25.7; 309) | 9.8(8.2-11.7; 132) | 18.2(16.0-20.6; 244) | 2.4(1.6-3.4; 32) |
|  | 2014 | 1402 | 41.2(37.9-44.7; 578) | 22.3(19.9-24.9; 312) | 14.3(12.4-16.5; 201) | 19.0(16.8-21.5; 267) | 2.8(2.0-3.8; 39) |
|  | 2015 | 1627 | 50.3(46.9-53.8; 818) | 18.9(16.8-21.1; 307) | 13.3(11.6-15.2; 217) | 15.2(13.3-17.2; 247) | 2.1(1.4-2.9; 34) |
|  | 2016 | 1425 | 49.8(46.2-53.6; 709) | 16.0(14.0-18.2; 228) | 15.9(13.9-18.1; 226) | 15.1(13.1-17.2; 215) | 2.9(2.1-4.0; 42) |
|  | 2017 | 1563 | 49.8(46.3-53.4; 778) | 14.4(12.6-16.4; 225) | 19.2(17.1-21.5; 300) | 14.6(12.8-16.6; 228) | 1.7(1.1-2.5; 27) |
|  | 2018 | 1645 | 47.8(44.5-51.2; 786) | 13.7(11.9-15.6; 225) | 23.1(20.8-25.5; 380) | 12.0(10.4-13.8; 198) | 2.9(2.1-3.8; 47) |
|  | 2019 | 1610 | 47.3(44.0-50.7; 761) | 9.9(8.4-11.5; 159) | 28.5(26.0-31.2; 459) | 12.0(10.4-13.9; 194) | 1.7(1.1-2.4; 27) |
| Gender | Male | 4086 | 47.3(45.2-49.4; 1932) | 17.4(16.2-18.8; 712) | 18.2(16.9-19.5; 743) | 13.4(12.3-14.5; 546) | 3.3(2.8-3.9; 135) |
|  | Female | 9747 | 45.4(44.1-46.8; 4429) | 21.1(20.2-22.0; 2053) | 13.9(13.2-14.6; 1353) | 17.3(16.5-18.1; 1684) | 2.0(1.7-2.3; 193) |
| Age group | 6-11 years | 490 | 38.4(33.1-44.3; 188) | 4.9(3.1-7.3; 24) | 19.0(15.3-23.3; 93) | 35.5(30.4-41.2; 174) | 1.4(0.6-2.9; 7) |
|  | 12-14 years | 2282 | 54.8(51.8-57.9; 1250) | 9.1(7.9-10.4; 208) | 15.6(14.0-17.3; 355) | 19.4(17.6-21.3; 442) | 0.8(0.5-1.3; 19) |
|  | 15-17 years | 11061 | 44.5(43.3-45.8; 4923) | 22.9(22.0-23.8; 2533) | 14.9(14.2-15.6; 1648) | 14.6(13.9-15.3; 1614) | 2.7(2.4-3.1; 302) |
| Deprivation | Least deprived | 2276 | 48.9(46.1-51.9; 1113) | 15.9(14.4-17.7; 363) | 15.2(13.6-16.9; 346) | 17.4(15.7-19.2; 395) | 2.1(1.5-2.7; 47) |
|  | 2 | 2006 | 45.3(42.4-48.4; 909) | 19.1(17.3-21.2; 384) | 15.4(13.7-17.2; 309) | 17.8(16.0-19.8; 358) | 1.6(1.1-2.3; 33) |
|  | 3 | 2488 | 44.0(41.4-46.7; 1094) | 21.7(19.9-23.6; 539) | 15.0(13.5-16.6; 374) | 17.2(15.6-18.9; 427) | 2.0(1.5-2.7; 51) |
|  | 4 | 2844 | 46.2(43.7-48.7; 1313) | 20.1(18.5-21.8; 572) | 15.3(13.9-16.8; 436) | 15.6(14.2-17.1; 443) | 2.5(1.9-3.1; 70) |
|  | Most deprived | 3851 | 45.7(43.6-47.9; 1760) | 21.8(20.3-23.3; 839) | 15.1(13.9-16.3; 580) | 14.0(12.8-15.2; 538) | 3.1(2.6-3.7; 120) |
| 1. groups not mutually exclusive, participants can have multiple prescriptions and may be counted more than once 2. Other SSRIs not shown due to small numbers | | | | | | | |
